# Supplementary material for: Multiple performance peaks for scale-biting in an adaptive radiation of pupfishes
Source: bioRxiv. 2023 Dec 23:2023.12.22.573139. Preprint. [Version 1] doi: 10.1101/2023.12.22.573139 (PMC10769438; doi:10.1101/2023.12.22.573139)
Supplement: Supplement 1 [file NIHPP2023.12.22.573139v1-supplement-1.pdf]

bioRxiv preprint doi: https://doi.org/10.1101/2023.12.22.573139; this version posted December 23, 2023. The copyright holder for this preprint (which was not certified by peer review) is the author/funder, who has granted bioRxiv a license to display the preprint in perpetuity. It is made available under aCC-BY 4.0 International license.

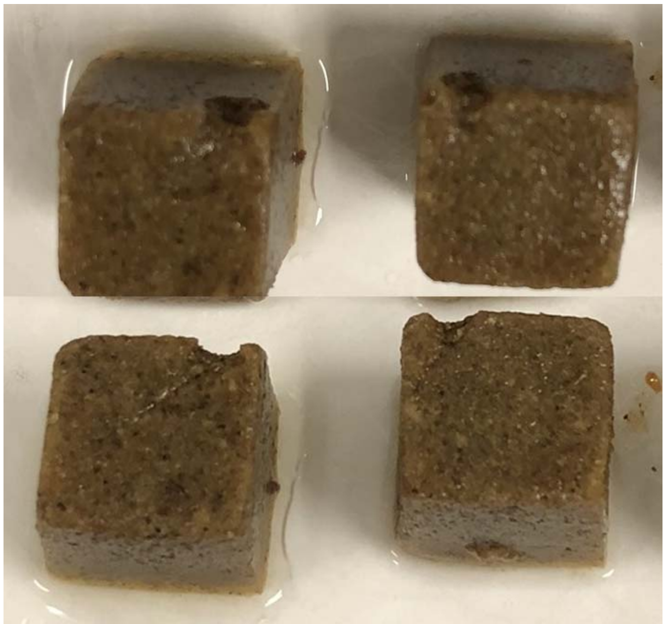

Fig. S1 Top-down and side views of typical single bites from Repashy gelatin cubes. Both bites pictured were classified as edge bites, the most common type of bite recorded.

bioRxiv preprint doi: https://doi.org/10.1101/2023.12.22.573139; this version posted December 23, 2023. The copyright holder for this preprint (which was not certified by peer review) is the author/funder, who has granted bioRxiv a license to display the preprint in perpetuity. It is made available under aCC-BY 4.0 International license.

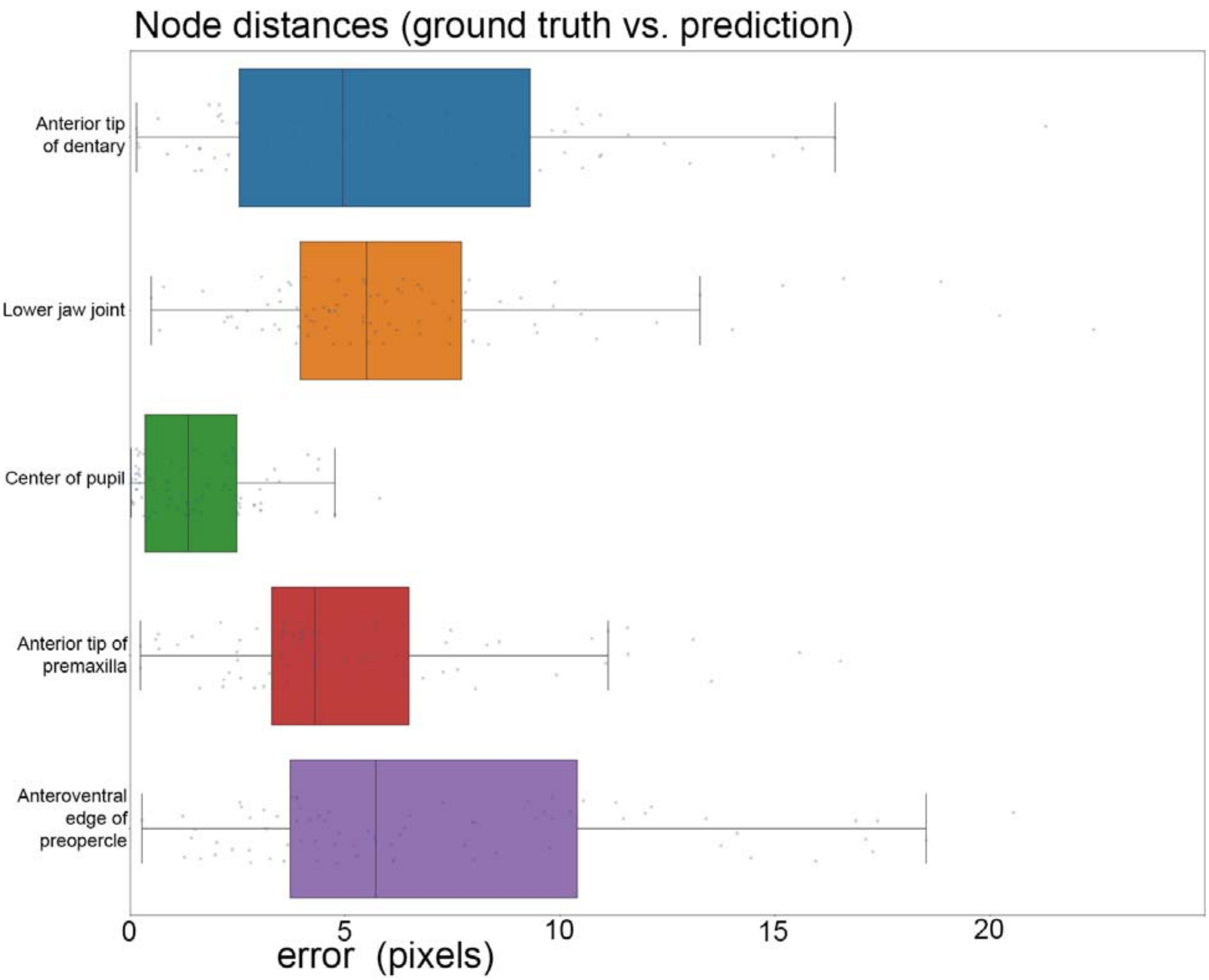

Fig. S2 Error distribution for each landmark in our best performing model. Trained model was based on 815 labeled frames across 100 high-speed videos. We used the multi-animal bottom-up unet model with a receptive field of 156 pixels, max stride of 32 pixels, batch size of 3, input scaling of 0.75, and validation fraction of 0.1. The mean distance between labeled data and inferred landmark positions was 5.80 pixels. Precision obtained was 0.990 based on four false positives, five false negatives, and 397 true positives in the model summary output from SLEAP.
